# Tumor Angiogenesis Therapy Using Targeted Delivery of Paclitaxel to the Vasculature of Breast Cancer Metastases

**DOI:** 10.1155/2014/865732

**Published:** 2014-12-07

**Authors:** Shijun Zhu, Walter Kisiel, Yang J. Lu, Lars C. Petersen, John M. Ndungu, Terry W. Moore, Ernest T. Parker, Aiming Sun, Dennis C. Liotta, Bassel F. El-Rayes, Daniel J. Brat, James P. Snyder, Mamoru Shoji

**Affiliations:** ^1^Department of Hematology and Medical Oncology, Winship Cancer Institute, Emory University, 1365 Clifton Road, NE, Atlanta, GA 30322, USA; ^2^Department of Pathology, University of New Mexico Health Sciences Center, Albuquerque, NM 87131, USA; ^3^Biopharmaceuticals Research Unit, Novo Nordisk A/S, 2760 Måløv, Denmark; ^4^Department of Chemistry, Emory University, 1515 Dickey Drive, Atlanta, GA 30322, USA; ^5^Department of Medicinal Chemistry and Pharmacognosy and UIC Cancer Center, University of Illinois at Chicago, Chicago, IL 60612, USA; ^6^Department of Pathology, Winship Cancer Institute, Emory University, 1365 Clifton Road, NE, Atlanta, GA 30322, USA

## Abstract

Breast cancer aberrantly expresses tissue factor (TF) in cancer tissues and cancer vascular endothelial cells (VECs). TF plays a central role in cancer angiogenesis, growth, and metastasis and, as such, is a target for therapy and drug delivery. TF is the cognate receptor of factor VIIa (fVIIa). We have coupled PTX (paclitaxel, also named Taxol) with a tripeptide, phenylalanine-phenylalanine-arginine chloromethyl ketone (FFRck) and conjugated it with fVIIa. The key aim of the work is to evaluate the antiangiogenic effects of PTX-FFRck-fVIIa against a PTX-resistant breast cancer cell line. Matrigel mixed with VEGF and MDA-231 was injected subcutaneously into the flank of athymic nude mice. Animals were treated by tail vein injection of the PTX-FFRck-fVIIa conjugate, unconjugated PTX, or PBS. The PTX-FFRck-fVIIa conjugate significantly reduces microvessel density in matrigel (*p* < 0.01–0.05) compared to PBS and unconjugated PTX. The breast cancer lung metastasis model in athymic nude mice was developed by intravenous injection of MDA-231 cells expressing luciferase. Animals were similarly treated intravenously with the PTX-FFRck-fVIIa conjugate or PBS. The conjugate significantly inhibits lung metastasis as compared to the control, highlighting its potential to antagonize angiogenesis in metastatic carcinoma. In conclusion, PTX conjugated to fVIIa is a promising therapeutic approach for improving selective drug delivery and inhibiting angiogenesis.

## 1. Introduction

*Scope*. The objective is to selectively deliver a highly toxic drug to tumor cells and the related tumor vascular endothelium. The target is tissue factor (TF), a membrane bound protein aberrantly expressed on cancer cells and their endothelia. The drug carrier is factor VIIa (fVIIa), the natural ligand of TF. fVIIa is transformed to its competitive inhibitor by conjugation with paclitaxel-FFRck, resulting in paclitaxel- (PTX-) FFRck-fVIIa. The conjugation prevents thromboembolic complications associated with fVII administration and provides an effective antiangiogenic approach that targets TF-expressing endothelia and cancers.

Tissue factor (TF) is a 47 kDa transmembrane glycoprotein receptor of* factor VII/VIIa (fVIIa)*, a critical regulator of tissue hemostasis and one of the body's most potent procoagulants [1]. Under normal conditions, TF is expressed by stromal cells, outer blood vessel layers (smooth muscle and adventitia), but not by vascular endothelial cells (VECs) (the inner most layer). Injury of the vascular wall causes TF to bind to its highly specific activating ligand,* fVIIa* from the plasma to initiate thrombosis, with subsequent hemostasis by generating a thrombin/fibrin deposit [2, 3]. In this context, angiogenesis is a crucial process for tumor progression and metastasis [4]. In a study of 328 patients with breast cancer, tumor angiogenesis was assessed by microvessel density (MVD) to show that the prognosis of patients with MVD greater than 100 microvessels/mm^2^ in a microscope field was significantly worse (*p* < 0.0001) than the prognosis of patients with lower MVD [5]. Nawroth and colleagues demonstrated that transfection of the cDNA of full length TF (flTF) in a sense orientation accelerated angiogenesis and tumor growth, whereas that in the antisense cDNA orientation decreased angiogenesis and tumor growth [6]. Phosphorylation of the serine residues on the cytoplasmic domain of flTF by protein kinase C (PKC) [7] leads to VEGF production. Furthermore, deletion of the flTF cytoplasmic domain impairs VEGF production in melanomas [8]. Clearly, the aberrant production and activation of TF are a deleterious factor in the angiogenic process.

A variety of cancers show increased expression of TF by neoplastic cells, and a direct correlation between TF levels and tumor grade has been noted for multiple tumor types [9–12]. TF has also been shown to be aberrantly expressed by the VECs of cancer tissue, a highly pathologic finding that promotes thrombosis. In breast cancers, TF is abnormally expressed by the endothelium in cancer lesions, which is highly pathologic as it promotes thrombosis.

On the other hand, it also provides a means to selectively target tumor tissue [9–12]. The aberrant expression of TF on tumor VECs induced by VEGF and its central role in angiogenesis provides a solid rationale for drug delivery [9, 13–16], including targeting TF for neovascular-targeted therapy such as immunotherapy and photodynamic therapy [17, 18].

Given the high affinity of fVIIa for TF, an enzymatically inactivated form was developed by conjugating phenylalanine-phenylalanine-arginine chloromethyl ketone (FFRck) to the enzymatic site of fVIIa [19–21]. The resulting FFRck-fVIIa is unable to initiate blood clotting, yet it has a 5-fold greater binding affinity to TF than native fVIIa [22]. The potential of this particular delivery method can be maximized by inserting highly potent, but also very toxic, anticancer drugs such as paclitaxel (PTX) into tumor cells specifically to reduce toxic effects distinct from the target area.

The present study evaluates the* in vivo* selective drug delivery and antiangiogenic effects of PTX conjugated to FFRck-fVIIa against a PTX-resistant breast cancer cell line. The results suggest that the drug-conjugate is directed at endothelial and neoplastic cells. Thus, PTX-FFRck-fVIIa can block the vicious cycle of thrombosis, necrosis, hypoxia, VEGF secretion, tumor angiogenesis and invasion.

## 2. Materials and Methods

The human breast cancer cell line MDA-MB-231-luc-D3H1 containing firefly luciferase gene was purchased from Caliper Lifesciences. The human head and neck squamous carcinoma (SCC) cell line Tu212 was provided by Dr. Dong M. Shin (Emory University, Atlanta, GA). Eagle's MEM medium with Earle' salt containing 2 mM L-glutamine (Cat. number 10-010-CV), DMEM/F12 (1 : 1) (Cat. number 12-719F), and inactivated fetal bovine serum (FBS) (Cat. number 26140-079) were purchased from Mediatech, Inc. (Herndon, VA 20171), Lonza Group Ltd. (Basel, Switzerland), and GIBCO (Grand Island, NY), respectively. Paclitaxel was purchased from Natland International Corporation (Research Triangle Park, NC). Recombinant factor VIIa and FFRck-fVIIa (XXV-22W, 2.65 mg protein/mL) were kindly provided by Dr. Walter Kisiel and Dr. Lars C. Petersen (University of New Mexico and Novo Nordisk, resp.). Factor VII-deficient plasma (Product number 0700) was purchased from George King Bio-Medical, Inc. (Overland Park, KS 66210-4192). Reagent 1, STA Neoplastine Cl (freeze-dried thromboplastin prepared from rabbit cerebral tissue) (Cat. number 00605), and Reagent 2, solvent containing calcium and sodium azide (Cat. number 00666), were purchased from Diagnostica Stago, Inc. (Parsippany-Troy Hills, NJ 07054). Matrigel (Cat. number 356231, deficient in growth factors) was purchased from BD Biosciences (San Jose, CA). Recombinant human VEGF-165 (VEGF-A) (10 *μ*g/vial, Catalog number 293 VE) was purchased from R & D, Minneapolis, MN. Antibodies to vWF (von Willebrand factor) (Abcam, Catalog number ab9378), rabbit anti-mouse tissue factor antibody (American Diagnostica Inc. Catalog number 4515), and anti-paclitaxel antibody (Novos Biologicals Catalog number NBP1-05003) were purchased from Abcam (ab9378), American Diagnostica Inc. (ab4515), and Novos Biologicals, respectively. Kits for immunohistochemical staining were purchased from Abcam HRP/DAB (ABC) Detection IHC kit (ab64264). Female athymic nude mice (nu/nu) were purchased from Harlan (Indianapolis, IN).

### 2.1. Cell Culture

MDA-MB-231-luc-D3H1 breast cancer cells (triple-negative for ER, PR, and Her2) and Tu212 human squamous carcinoma cells (SCC) were maintained in Eagle's MEM medium with Earle' salt containing 2 mM L-glutamine and DMEM/F12 (1 : 1), respectively, containing 10% heat inactivated FBS, penicillin (100 units/mL), streptomycin (100 *μ*g/mL), 0.1 mM nonessential amino acid at 37°C, and 5% CO_2_/95% air in a humidified atmosphere.

### 2.2. Synthesis of Paclitaxel- (PTX-) FFRck and Conjugation of PTX-FFRck to fVIIa

The tripeptide phenylalanine-phenylalanine-arginine chloromethyl ketone (FFRck) group is bound in an irreversible reaction to histidine 193 in the catalytic domain of fVIIa, inactivating its serine protease activity. The FFRck-fVIIa functions as a competitive inhibitor of fVIIa to tissue factor (TF) and has no coagulant activity [22]. PTX was chemically coupled to the first phenylalanine of the chloromethyl ketone (FFRck). The resulting PTX-FFRck was then incubated together with fVIIa under gentle stirring at 4°C overnight to form PTX-FFRck-fVIIa conjugate as previously described [23].

**Scheme 1:**
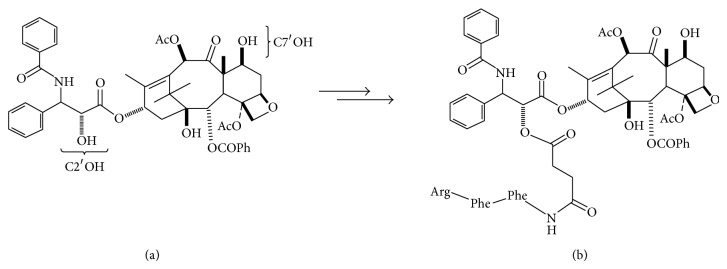
Paclitaxel (PTX). (a) At left the locations of C2′OH and C7′OH are labeled; (b) at right, the C2′OH moiety has been coupled with a glutamic acid linker and the corresponding Phe-Phe-Arg tripeptide to give C2′-PTX-FFRck.

The conjugation procedure is essentially the same as that described for conjugation of EF24 [24–27]. Briefly, C2′-PTX-FFRck in 100% DMSO was added dropwise to the fVIIa (MW 50,000) solution in a molar ratio of 3 : 1 at room temperature for 1-2 h while stirring, followed by gentle stirring at 4°C overnight. C7′-PTX-FFRck is less active than C2′-PTX-FFRck. C2′ and C7′ indicate the location of hydroxyl groups (OH) on PTX to which PTX-FFRck is conjugated and illustrated in Scheme 1 [23]. The unconjugated excess PTX-FFRck (FW: 1,500) was removed by exhaustive dialysis at 4°C using a dialysis membrane with a pore size of 13,000–15,000 MW to exclude molecules below 13,000 MW in 4 L of 10 mM Tris-HCl, pH 7.5, with several changes of the buffer every 12 h for several days to ensure all unbound PTX-FFRck is dialyzed out.

### 2.3. Determination of Residual fVIIa Activity of the PTX-FFRck-fVIIa Conjugate

The PTX-FFRck-fVIIa conjugate, containing trace amounts of fVIIa, was diluted to 10^−4^ and 10^−5^ for the assay. Residual fVIIa activity in the PTX-FFRck-fVIIa conjugate was then measured according to the manufacturer's instruction (Diagnostica STAGO, Parsippany, NJ) using an ST Art 4 instrument (Diagnostica STAGO, Asnieres-sur-Seine, France). A standard fVIIa curve was plotted as log U/mL versus clotting time for linear regression, and residual fVIIa was determined from the standard curve.

### 2.4. Inhibition by the PTX-FFRck-fVIIa Conjugate of MDA-MB-231 Cells and VEGF-A-Induced Angiogenesis in Matrigel Plugs in Mice

Prior to testing the efficacy on lung metastasis, a matrigel experiment was performed in mice to assess the inhibition of angiogenesis by the PTX-FFRck-fVIIa conjugate induced by MDA-MB-231 cells and VEGF-A. MDA-MB-231 cells produce high levels of both VEGF and TF [9, 11]. A matrigel level of 0.6 mL/mouse containing 600 ng of VEGF-A and 0.6 × 10^6^ MDA-MB-231-luc-D3H1 cells was implanted subcutaneously in the flanks of female athymic nude mice. On days 13, 14, and 15 after matrigel implantation, (a) 0.1 mL of PTX-FFRck-fVIIa conjugate (containing 1.3 *μ*g PTX equivalent/mouse = 65 *μ*g PTX/kg body weight), (b) 0.1 mL of unconjugated PTX (1.3 *μ*g/mouse), or (c) 0.1 mL of PBS (vehicle) was administered intravenously into the tail vein. On day 19, matrigel plugs were collected, and paraffin embedded. Five mice per each regimen were treated. Microvessel density (MVD) per whole matrigel was determined using hematoxylin and eosin (H&E) stained slides counted by a blinded examiner.  Figure 2(a) is a representative schema of the treatment plan, and Figure 2(b) shows microvessel numbers in matrigel in each treatment.

### 2.5. Neutral Red Dye Cell Viability Assays

The efficacy of PTX-FFRck-fVIIa against various cell lines was tested* in vitro* using neutral red (NR) dye cell viability assays as previously described [28]. Cell viability following 48 h drug treatment was assayed by using the NR dye. The latter is taken up by viable cells. Briefly, at the termination of culture, existing medium was removed and 200 *μ*L of fresh, warm medium containing 50 *μ*g of NR/mL was added to each well in a 96-well plate. Cells were incubated at 37°C for 30 min, followed by two washes with 200 *μ*L of PBS. The NR taken up by cells was dissolved by adding 200 *μ*L of 0.5 N HCl containing 35% ethanol. Then, the amount of the dye in each well was read at 570 nm with an EL800 Universal Microplate Reader (Bio-Tek Instruments, Inc., Tustin, CA). Each experiment was performed in triplicate and analyzed by Student's *t*-test. Dose-response curves of PTX and PTX-FFRck-fVIIa were determined using MDA-MB-231 breast cancer cells and Tu212 SCC.

### 2.6. Immunohistochemical Staining of TF of VECs in Lung Metastasis and of PTX in Breast Cancer Xenografts in the Lung

VECs were stained using anti-von Willebrand Factor (vWF) antibody in 1 : 100 dilution. TF was stained using anti-mouse TF antibody in 1 : 100 dilution. PTX in the lungs was stained using paclitaxel antibody. The Mouse and Rabbit Specific HRP/DAB detection IHC kit (Abcam, ab64264) was used according to manufacturer's instructions. H&E staining was used for histological diagnosis.

### 2.7. Lung Metastasis (Colonization)

Lung metastases of breast cancer were developed by injecting i.v. the MDA-MB-231-luc-D3H1 cells (2 × 10^6^ cells/0.1 mL/mouse) containing luciferase gene into the tail vein of athymic nude mice. Tumor cells are stuck in the capillaries before the first pass to the heart and randomly distributed in the lungs but no other organs. Animals were treated with PTX-FFRck-fVIIa conjugate (1.3 *μ*g PTX in PTX-FFRck-fVIIa/0.1 mL, 20 g body weight) or PBS (0.1 mL) i.v. on days 10, 11, 14–18, 23–26, and 28 following tumor cell inoculation (*n* = 10, each). Schematic representation of the experiment is presented in Figure 4. To assess the therapeutic response, we followed the change of the luciferase activity on days 7, 22, 29, 36, and 43 following inoculation using the Xenogen box (Caliper Lifesciences, Inc.). All protocols for animal studies were reviewed and approved by the Institutional Animal Care and Use Committee at Emory University. To perform bioluminescence imaging of tumors in the lungs, the animals were anesthetized by intraperitoneal injection of 0.1 mL of a mixture of 0.8 mL of ketamine from a vial containing 10 mg/mL, 0.1 mL of xylazine from a vial containing 100 mg/mL, and 0.9 mL of sterile water.

### 2.8. Statistical Analysis

Data obtained were analyzed using one-way ANOVA. Results are considered to be significantly different when *p* values are less than 0.05.

## 3. Results

### 3.1. The PTX-FFRck-fVIIa Conjugate as an fVIIa Inhibitor

**Figure 1:**
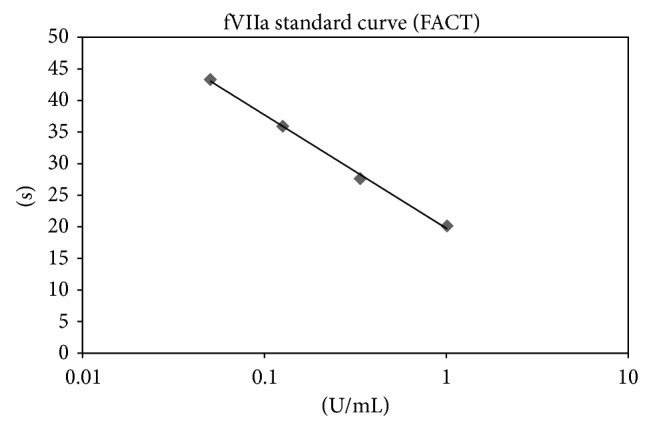
Standard curve of the fVIIa activity. A standard curve of the fVIIa activity was plotted as log U/mL versus clot time (in seconds) for linear regression. Units/mL for the unknowns were determined from the standard curve.

Measurement of the fVIIa activity of the PTX-FFRck-fVIIa preparation revealed that the residual clot activity was less than 5% that of the unconjugated fVIIa control, indicating that approximately 95% fVIIa was bound by PTX-FFRck and converted to PTX-FFRck-fVIIa, a competitive inhibitor of fVIIa [23] (Table 1 and Figure 1).

### 3.2. Inhibition of MDA-MB-231 Cells and VEGF-A-Induced Angiogenesis in Matrigel Plugs in Mice by the PTX-FFRck-fVIIa Conjugate

**Figure 2:**
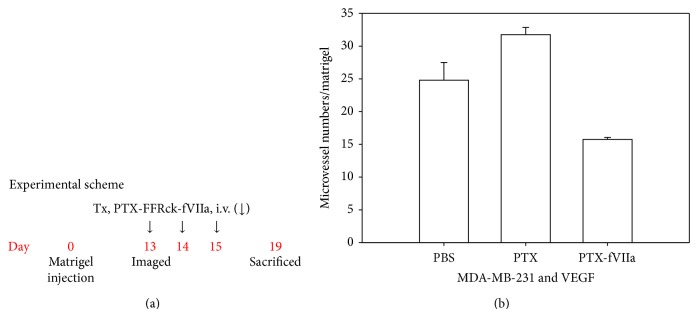
PTX-FFRck-fVIIa inhibits angiogenesis in matrigels. Matrigel containing MDA-MB-231 cells and VEGF-A was implanted s.c. in the flanks of female athymic nude mice. On days 13, 14, and 15 after the matrigel implantation, PBS, PTX alone, and PTX-FFRck-fVIIa conjugate were administered intravenously (i.v.) into the tail vein. On day 19, matrigel plugs were collected, and microvessel density (MVD) per whole matrigel stained with hematoxylin & eosin was counted. (a) Experimental scheme. (b) Microvessel number in matrigel treated with PBS, PTX alone and PTX-FFRck-fVIIa (*n* = 5/regimen). The *p* values between PBS versus PTX, PBS versus PTX-FFRck-fVIIa, and PTX versus PTX-FFRck-fVIIa are *p* < 0.05, *p* < 0.05, and *p* < 0.01, respectively.

PTX-FFRck-fVIIa significantly reduced MVD as compared with PBS or unconjugated PTX alone in matrigels containing MDA-MB-231 cells and VEGF-A (Figure 2). PBS and PTX alone (in a dose equivalent to the amount of PTX in PTX-FFRck-fVIIa) did not reduce MVD. The *p* values of the MVD in matrigel treated with PBS, PTX alone, and PTX-FFRck-fVIIa (*n* = 5/regimen) between PBS versus PTX, PBS versus PTX-FFRck-fVIIa, and PTX versus PTX-FFRck-fVIIa are *p* < 0.05, *p* < 0.05, and *p* < 0.01, respectively.

### 3.3. Cytotoxic Activity of PTX-FFRck-fVIIa against MDA-MB-231 Breast Cancer Cells and Tu212 SCC Cells* In Vitro*

**Figure 3:**
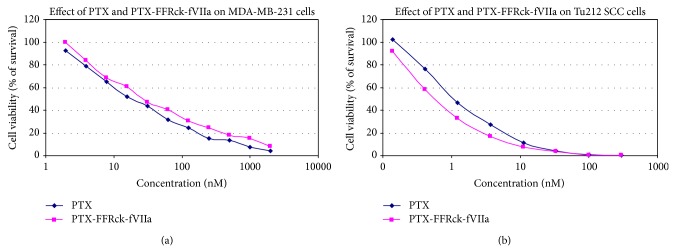
Cytotoxic activity of PTX-FFRck-fVIIa against MDA-MB-231 breast cancer cells and Tu212 SCC cells* in vitro*. PTX and PTX-FFRck-fVIIa require over 1,000 nM to kill MDA-MB-231 cells but between 10 and 100 nM to kill Tu212 cells 100%, respectively.

PTX and PTX-FFRck-fVIIa require over 1,000 nM to kill MDA-MB-231 cells but between 10 and 100 nM to kill Tu212 cells 100%, respectively. Hence, MDA-MB-231 cells are more resistant to PTX and PTX-FFRck-fVIIa than Tu212 SCC (Figure 3). It is unclear why Tu212 cells are more sensitive to PTX than MDA-MB-231 cells. PTX is slightly better than PTX-FFRck-fVIIa to MDA-MB-231 cells* in vitro*, but PTX-FFRck-fVIIa is clearly more efficacious than PTX at the same concentration because the PTX-FFRck-fVIIa conjugate binds TF on VECs* in vivo* but PTX alone does not, as shown in Figure 2.

### 3.4. PTX-FFRck-fVIIa Inhibits Tumor Angiogenesis and Attenuates Growth of Drug-Resistant Breast Cancer Xenografts in the Lung

The experiments were performed to demonstrate that antitumor angiogenesis therapy may inhibit a drug-resistant tumor. MDA-MB-231 cells are relatively resistant to PTX-FFRck-fVIIa (Figure 3). However, the PTX-FFRck-fVIIa conjugate demonstrates antitumor angiogenesis activity, whereas PBS and PTX alone do not (Figure 2). Therefore, we compared the efficacy of PTX-FFRck-fVIIa and PBS* in vivo*.

**Figure 4:**
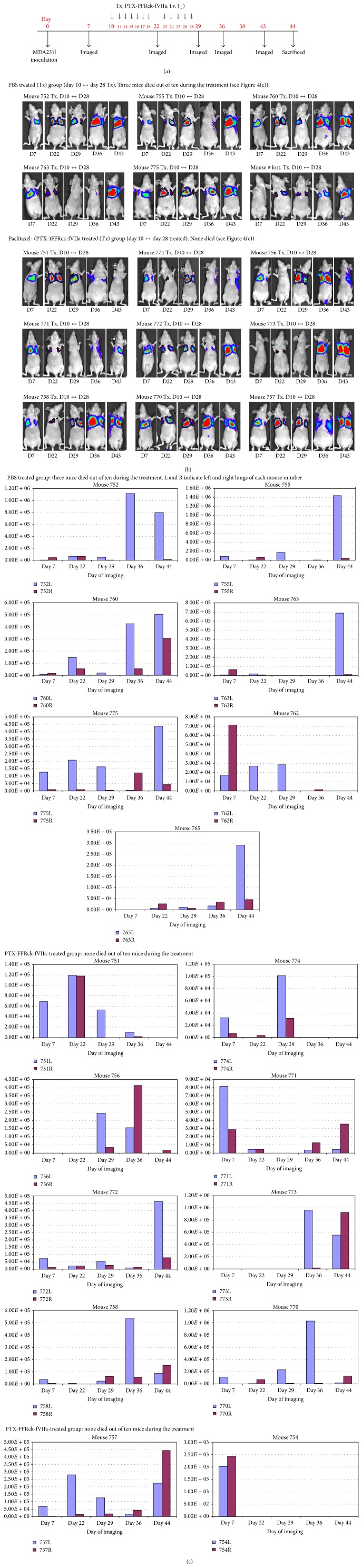
PTX-FFRck-fVIIa reduced breast cancer lung metastasis by inhibiting tumor angiogenesis. On day 0, human breast cancer MDA-MB-231-luc-D3H1 cells containing luciferase gene were injected i.v. into the tail vein of female athymic nude mice. Twelve treatments with PTX-FFRck-fVIIa and PBS were administered i.v. into the tail veins on days 10, 11, 14–18, 23–26, and 28 following tumor cell inoculation (*n* = 10 mice/regimen). Therapeutic response of the tumor xenografts was followed by imaging the luciferase activity. (a) Experimental scheme. (b) Images of the luciferase activity of breast cancer xenografts in the lungs. (c) Quantitation of the luciferase activity of (b).

Cells were intravenously injected into ten mice per each regimen. Three mice in the PBS-treated group died before completion of the experiments, possibly due to excessive tumor growth, whereas none died in the PTX-FFRck-fVIIa-treated group. In the PBS group, tumor xenografts grew larger after completion of treatment on day 28 in 6 of the 7 mice. In the PTX-FFRck-fVIIa group, tumor growth in 7 of the 10 mice was inhibited during the treatment period between day 10 and day 28 and/or thereafter (Figure 4).

### 3.5. Immunohistochemical (IHC) Staining of TF on Vascular Endothelial Cells in Lung Metastasis

**Figure 5:**
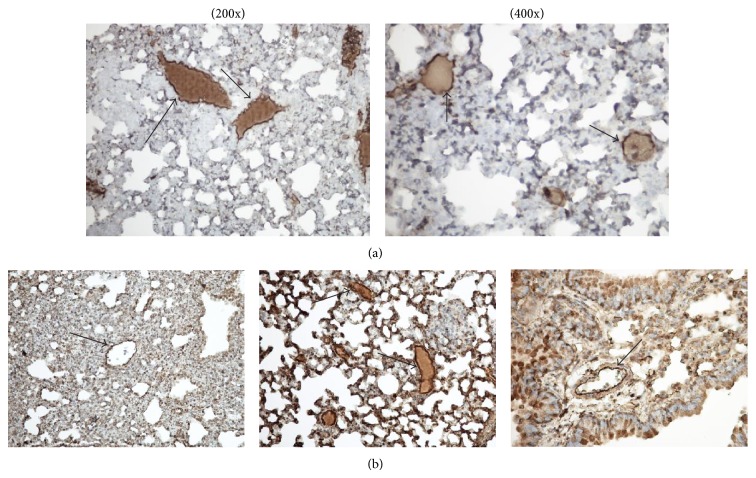
TF and vWF stains of endothelium in breast cancer lung metastases. (a) TF staining using an anti-mouse TF antibody (arrows indicate a single layer of endothelial cell stained darker brown). Photographs are 200x and 400x, respectively. (b) vWF staining using anti-vWF antibody (arrows indicate a single layer of endothelial cell stained darker brown). All photographs are 200x.

TF expressed on the endothelium in the cancer milieu is the target of drug delivery by its ligand fVIIa used as a drug carrier. In Figure 5(a) TF is expressed on the tumor endothelium (single layer), which coincides with an endothelial marker, vWF (Figure 5(b)) in the lung xenografts of breast cancer.

### 3.6. Paclitaxel Is Localized in Breast Cancer Tumor Xenografts in the Lung

**Figure 6:**
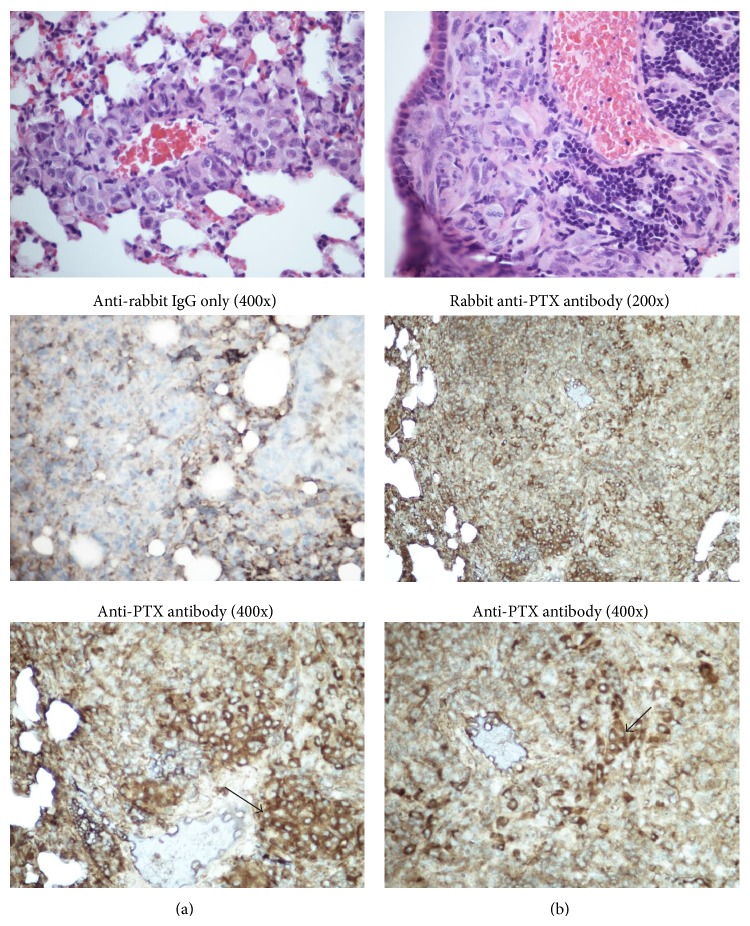
Paclitaxel (PTX) staining in MDA-MB-231 lung metastasis. The H&E stain demonstrates human breast cancer MDA-MB-231 cells surrounding a blood vessel in the lung (a) and mononuclear cells adjacent to a blood vessel that are much smaller than breast cancer cells (b). The IHC stains demonstrate that the cytoplasm of rare scattered tumor cells is faintly stained brown by anti-rabbit IgG immunoglobulin, in contrast to the cells stained with anti-PTX antibody, which shows intense brown staining of the cytoplasm.

To confirm the delivery of PTX by PTX-FFRck-fVIIa to the metastatic tumors, localization of PTX was determined by IHC staining using anti-PTX antibody. In Figure 6, the H&E stain was included to demonstrate the presence of tumor, in this case predominantly around blood vessels (Figures 6(a) and 6(b)). The H&E stain demonstrates that human breast cancer MDA-MB-231 cells are enlarged cells, with atypical nuclei and numerous mitoses around the vasculature of the lung (Figure 6(a)). There are also mononuclear inflammatory cells adjacent to blood vessel that are much smaller than breast cancer cells (Figure 6(b)).

The cytoplasm of rare scattered tumor cells is faintly stained brown by anti-rabbit IgG immunoglobulin, in contrast to the cells stained with anti-PTX antibody, which shows intense brown staining of the cytoplasm. The tumor grows as a solid mass within the lung, with almost no viable bronchiolar spaces present, except for one region in which bronchiolar spaces are pushed to the periphery (Figure 6; panel magnified 200x). We stained PTX in lung tissues harvested two weeks after PTX-FFRck-fVIIa was discontinued.

## 4. Discussion

In this paper, we demonstrate that PTX-FFRck-fVIIa delivers PTX selectively to TF-expressing vascular endothelium and tumor metastases in the lungs. Conjugating PTX to factor VIIa allows for delivery of the drug to TF-expressing VECs. Since only tumor VECs express TF on the luminal surface, this approach provides a selective delivery of PTX to tumor VECs. The cytotoxic effects of PTX can inhibit VECs leading to suppression of angiogenesis and tumor growth. In addition, the drug carrier FFRck-fVIIa is the competitive inhibitor of factor VIIa and is expected to inhibit intravascular thrombosis caused by TF-expressing tumor vasculature and circulating TF released from tumor. Thus, the drug-conjugate not only is directed at endothelial and neoplastic cells but also blocks the vicious cycle of thrombosis, necrosis, hypoxia, VEGF secretion, tumor angiogenesis and invasion.

A major concern in patients with advanced cancer is the increased risk of thrombosis. Factor VIIa is a natural procoagulant and an activator of the coagulation cascade. FFRck-fVIIa was initially developed as a potential anticoagulant given that it inhibits the binding of factor VIIa to tissue factor. Clinical experience has shown that use of FFRck-fVIIa is safe [29, 30]. The main complications observed in clinical trials were related to slight increased risk of bleeding, which is expected in anticoagulant therapy. Therefore the use of FFRck-fVIIa as an approach for drug delivery is feasible in the clinical setting. Since the drug-conjugate specifically delivers drug to target cells where TF is concentrated, this approach achieves a cytotoxic effect at a lower drug concentration than required for an unconjugated drug. In turn this will limit the potential side effects of the cytotoxic agent. In this paper, we demonstrate that the conjugated PTX has greater anti-angiogenic activity than an equivalent amount of PTX using the matrigel experiment. This provides further support that targeted delivery may improve activity and lower toxicity for standard cytotoxic agents.

In many instances, targeted therapy is used to target a single signaling pathway. This is not the case for cancer cells and the endothelium in cancer. Targeting a single pathway has not proved to be effective for extended treatment, since tumor cells develop alternative signaling pathways to circumvent the inhibition by a single inhibitor or a combination of several single signaling pathway inhibitors [31–33]. Moreover, these signaling-based therapies exhibit the same frequency and severity of toxicities as traditional cytotoxic agents, the main difference being the nature of the side effects [34].

The targeting of PTX to TF-expressing VECs and neoplastic cells is a highly specific approach. TF is only expressed by pathologic blood vessels and is therefore an ideal target. The use of the high affinity factor VII ligand for TF as the targeting agent is employed to take advantage of one of the body's most specific protein interactions. It is anticipated that it will be generally useful for directing other therapies to TF-expressing cells.

## 5. Conclusions

The efficacy of antiangiogenesis therapy was tested against paclitaxel-resistant MDA-MB-231-luc-D3H1 breast cancer xenografts in the lung. Paclitaxel-FFRck-fVIIa was delivered to both TF-expressing tumor vasculature and tumors. The targeted delivery of the drug-conjugate was efficacious and suppressed tumor growth of the drug-resistant breast cancer xenografts. Treated animals did not demonstrate any bleeding tendency or signs of physical impairment. This approach may be useful for controlling drug-resistant tumor metastasis.

## Figures and Tables

**Table 1:** PTX-FFRck-fVIIa as a competitive inhibitor of factor VIIa.

Factor VIIa activity	Clotting time (in sec.)	% inhibition of fVIIa activity
fVIIa	14.3	0
PTX-FFRck-fVIIa	37.5	93.2
